# Investigation on the Stability of Derivative Melam from Melamine Pyrolysis under High Pressure

**DOI:** 10.3390/nano8030172

**Published:** 2018-03-18

**Authors:** Xiaohong Yuan, Kun Luo, Yingju Wu, Julong He, Zhisheng Zhao, Dongli Yu

**Affiliations:** 1 State Key Laboratory of Metastable Materials Science and Technology, Yanshan University, Qinhuangdao 066004, China; xhyuan_future@163.com (X.Y.); hiluokun@gmail.com (K.L.); wuyingjv@163.com (Y.W.); hjl@ysu.edu.cn (J.H.); 2 Hebei Key Laboratory of Microstructural Material Physics, School of Science, Yanshan University, Qinhuangdao 066004, China

**Keywords:** melamine, melam, high pressure, pyrolysis, s-triazine, g-C_3_N_4_

## Abstract

Although various kinds of carbon nitride precursors have been proposed, s-triazine-based structures are hardly reported because of their unfavorable energy, higher than that of heptazine-based ones. In this study, we investigate the thermal stability of s-triazine-based melam processed at a high pressure of 5 GPa and a temperature of 400–700 °C and complete the analyses of the composition and structure of the treated samples through X-ray diffraction (XRD), Fourier-transform infrared spectroscopy (FTIR), and elemental analyses (EA). Results show that melam can stably exist up to 600 °C at 5 GPa. XRD and FTIR analyses reveal that residual melamine can be pyrolyzed into melam as temperature increases from 400 °C to 600 °C at a high pressure, suggesting that melam may be purified through high-pressure pyrolysis. Further melam polymerization at a higher pressure is a promising strategy for the preparation of s-triazine-based carbon nitride precursors used for bulk carbon nitride synthesis.

## 1. Introduction

Liu [1], Cohen [2], and further theoretical studies [3,4,5,6] predicted that saturated *sp*^3^-hybridized carbon nitride compounds have a bulk modulus comparable to or larger than that of diamond. Since then, the synthesis of low-compressible C_3_N_4_ forms has been the main goal of studies related to superhard materials science [7]. In pioneering studies, high-pressure techniques have been proposed to transform a graphite-like carbon nitride (g-CN) into a covalent C–N solid with tetrahedral bonding similar to that observed in graphite-to-diamond process [2,3,8,9]. However, the proposed phases of C_3_N_4_ are hardly observed during high-pressure syntheses involving various polymeric forms of amorphous carbon nitride [10,11,12,13,14,15,16,17]. An ideal precursor is still expected to obtain high-density carbon nitrides through high-pressure and high-temperature techniques.

On the basis of the analysis of chemical bonding geometries, we can observe that layered C–N pyrolysate with an s-triazine (C_3_N_3_) unit should be preferred for the synthesis of high-density CN phases with a diamond-like *sp*^3^ bond. As such, the s-triazine derivative molecule melam, which is one type of melamine pyrolysis product, has been extensively investigated [18,19,20,21,22,23]. Melam is formed by linking two melamine molecules [20]. In melamine pyrolysis, melam is in a highly active intermediate state and has a strong tendency to be converted into melem. Hence, pure melam is difficult to obtain through pyrolysis at atmospheric pressure. The insolubility of melamine and its pyrolysis products also causes difficulties in purification [24,25].

The pressure-driven structural transitions of molecular crystals have been widely investigated [26,27,28,29]. As pressure increases, atomic distance decreases, thereby inducing condensed phases and strong interactions among molecules. These interactions gradually become stronger and closer to inner molecules [30], indicating that aggregation is more likely to occur than a decomposition reaction at a given pressure. However, an increase in the pressure of ammonia during this reaction slows down the polymerization of melamine to melam by enhancing the re-transition into melamine and the formation of melem [21,22]. Thus, the high-pressure structural transition of melam should be further investigated.

In this work, a sample primarily composed of melam was synthesized from pure melamine at 350 °C under nitrogen flow. A high pressure of 5 GPa was then applied to investigate the high-pressure phase transition of melam. The structures of the products at 5 GPa and at a temperature of up to 700 °C were studied through X-ray diffraction (XRD), infrared (IR) and Raman vibrational spectroscopies, and elemental analysis (EA). At 5 GPa, a phase transition from melam to melem did not occur before carbonization was obviously observed at 700 °C. The content of residual or unreacted melamine decreased as temperature increased from 400 °C to 600 °C at 5 GPa. Thus, high-pressure purification of melam and further polymerization of the s-triazine-based structure could be predicted in carbon nitride at high pressure.

## 2. Experimental Section 

### 2.1. Sample Preparation

A low-temperature (350 °C) deamination product (LTDP) was synthesized from pure melamine (1 g, >99%, Avocado) at 350 °C for 1 h in a quartz tube with a diameter of 35 mm under nitrogen flow in accordance with the procedure reported by B. Lotsch [20], E. Wirnhier [22], and Y. Kojima [12]. The procedure was repeated seven times to make the structural transition as complete as possible. The high-pressure pyrolysates of LTDP were obtained using a China-type CS 1B (6 × 8 MN) cubic anvil apparatus at 5 GPa and 400–700 °C.

### 2.2. Characterizations

An XRD (DMAX−2500/P, Rigaku, Japan) with Cu Kα radiation (Bragg-Brentano geometry, λ = 0.15406 nm, 40 kV, 200 mA) was used to characterize the samples. The Fourier-transform infrared (FTIR) spectra of the samples were obtained by using a Bruker Equinox 55 FTIR spectrometer (Bruker, Madison, WI, USA) with an attenuated total reflectance (ATR) mode. Measurements were conducted on KBr pellets (1 mg of sample; 100 mg of KBr; hand pressed with a capacity of 6 MPa) under ambient conditions between 400 and 4000 cm^−1^ in a transmission mode. The spectra were recorded at a resolution of 2 cm^−1^ and were averaged over 16 scans. Raman measurement was performed with a Renishaw InVia micro−Raman spectroscope (Renishaw, Wotton-under-Edge, Gloucestershire, UK) at laser radiations of 325, 532, and 633 nm. Prior to each experiment, the Raman shift was calibrated using the well-defined peak of silicon wafer at about 520 cm^−1^. After calibration, Raman spectra were recorded with acquisition time of 50 s and accumulations of 10 to improve the signal-to-noise ratio (SNR). EA was performed by using a C/H/N elemental analyzer (Vario MACRO cube EL, Elementar, Langenselbold, Germany).

## 3. Results and Discussion

**Figure 1:**
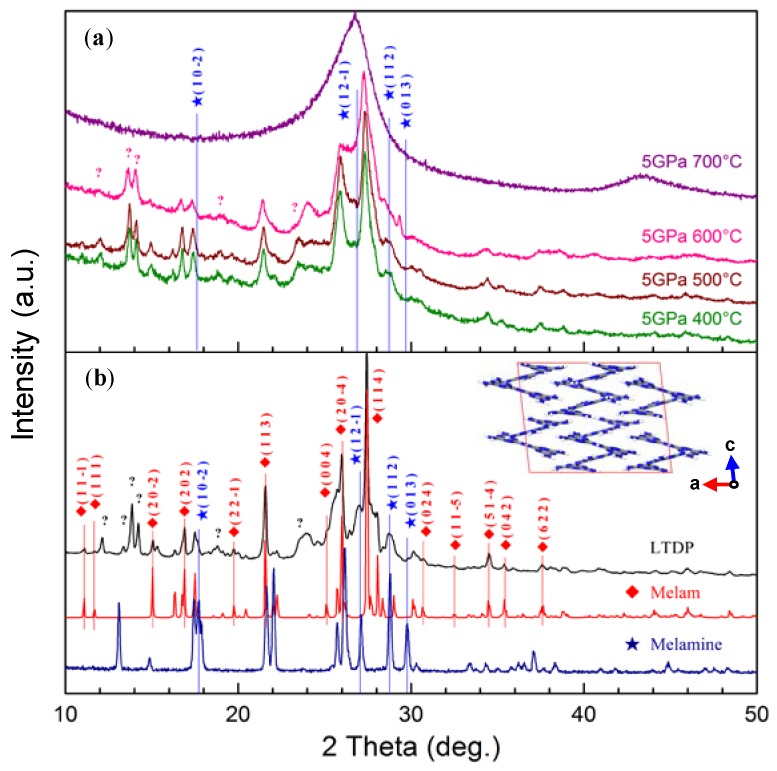
(**a**) XRD patterns of the samples treated at 5 GPa and different temperatures (400, 500, 600, and 700 °C); (**b**) XRD patterns of melamine, melam and LTDP sample. The inset in (**b**) Shows the crystal structure of melam.

The XRD spectra of melamine, melam [22], and LTDP are shown in Figure 1b. Comparing these spectra, we observe that most of the XRD peaks of LTDP correspond to the diffraction of previously reported melam [22]. Four small peaks at 17.73°, 27.10°, 28.76°, and 29.78° are assigned to the characteristic melamine (10$\bar{2}$), (12$\bar{1}$), (112), and (013) diffractions, respectively. XRD analysis reveals that LTDP is mainly composed of melam and a small amount of residual melamine. Six unknown diffraction peaks are located in the range of 12°–26° of the XRD spectrum, indicating that an unknown C–N or C–N–H structure may exist in the LTDP sample. The detailed XRD data of melamine and melam are listed in Tables S1 and S2, respectively.

Figure 1a illustrates that the structure of melam is effectively maintained in the treated LTDP sample at a high pressure of 5 GPa and temperatures of 400–600 °C. The pyrolytic behavior of melam at 5 GPa is different from that at atmospheric pressure. Two alternative schemes of melam transformation [20,23] have been proposed when melam is heated up to 600 °C at atmospheric pressure. In one of the schemes, melam transforms to melem and polymerizes into melon, which may further lose ammonia to form heptazine-based graphitic carbon nitride (g-C_3_N_4_). In the other route, melam is directly polymerized into heptazine-based g-C_3_N_4_. At 5 GPa, the structure of melam in the LTDP sample remains unchanged as the sample is heated to 600 °C. The intensity of melamine (10$\bar{2}$), (12$\bar{1}$), (112), and (013) diffraction peaks gradually decreases as temperature increases from 400 °C to 600 °C, indicating that melamine still polymerizes at a high pressure. The typical pungent smell of ammonia when the samples are taken out from the crucible also indicates that melamine pyrolysis occurs. Our analysis suggests that high pressure can stabilize the melam structure without affecting the further pyrolysis of melamine. Therefore, this mechanism may be a valuable method for the synthesis of pure melam using as a carbon nitride precursor.

The positions of the unknown peaks in the XRD spectrum of LTDP remain unchanged as the pyrolysis temperature increases, indicating that the decomposition of C–N or C–N–H products is also inhibited at a high pressure of 5 GPa. When the pyrolysis temperature reaches 700 °C, the appearance of the peaks at 26.7° and 43.5° corresponding to the diffraction peaks of graphite (002) and (101) planes suggests that the LTDP sample is graphitized.

**Figure 2:**
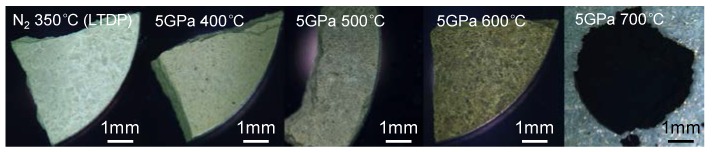
Optical image of LTDP and samples treated at 5 GPa and 400–700 °C.

As the pyrolysis temperature increases, the color of the LTDP samples gradually changes from yellowish to yellow at 5 GPa and 600 °C and black at 5 GPa and 700 °C (Figure 2). The reported band gap energies of g-C_3_N_4_ range from 2.4 to 2.85 eV depending on different preparation conditions [31,32,33,34,35]. It is smaller than that of melamine [36,37]. The Raman spectra at different laser radiations (Figure S1) shows that no graphite phase exists in the high-pressure-treated samples at temperatures below 700 °C. Thus, we tentatively conclude that changes in color are caused by the gradual decrease in melamine content with increasing temperature. Similar conclusions are also obtained from the XRD patterns of the samples.

**Figure 3:**
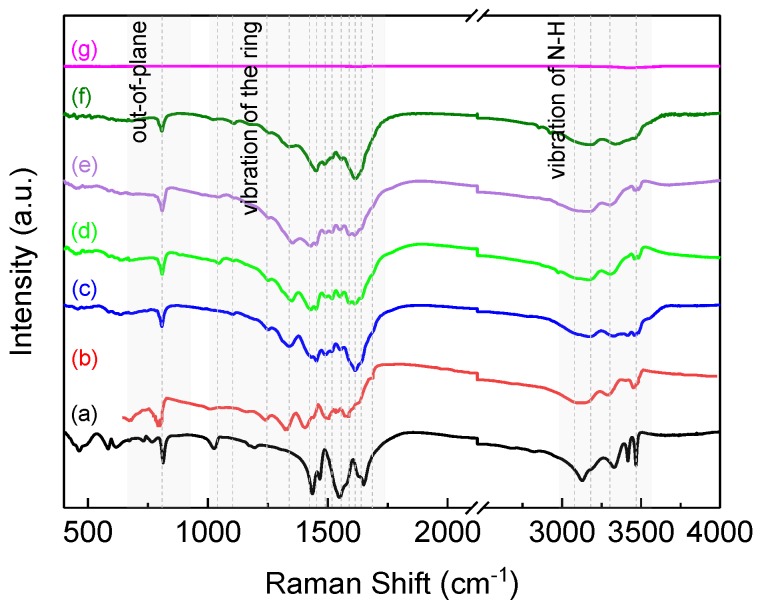
FTIR spectrum of melamine (**a**); melam (**b**); LTDP (**c**) and samples treated at 5 GPa and 400 °C (**d**); 500 °C (**e**); 600 °C (**f**); and 700 °C (**g**).

The FTIR spectra of melamine, melam [20], LTDP, and the sample obtained at 5 GPa and at different temperatures are shown in Figure 3. The detailed FTIR data of melam [20], melamine [38], and the samples are listed in Table 1. The following characteristic vibrations in the FTIR spectrum are given on the basis of the molecular structure of melam [20,22]: The band around 808 cm^−1^ can be attributed to the bend vibration mode of the sextant ring; the absorption bands at 1250 cm^−1^ are the NH_2_ shearing vibration modes; the peaks around 1340 cm^−1^ can be inferred for the C−N stretching modes; the absorption region between 1400 and 1700 cm^−1^ can be assigned to the coupling vibrations between NH_2_ shearing and C=N stretching modes, thus providing evidence for the structure of the triazine ring to be retained; and the peaks found between 3100 and 3500 cm^−1^ verify the presence of NH or NH_2_ groups. The spectral lines and data analysis confirm that the main phase in the LTDP sample is melam, and this observation is consistent with the results of XRD analysis. Comparing the FTIR spectra of high-pressure samples in Figure 3, we can detect the same trend of phase transition as the XRD pattern. At below 600 °C, the positions of the vibration peaks are almost the same as that of melam [21]. The basic s-triazine ring structure in the high-pressure samples remains undecomposed as the pyrolysis temperature increases. However, the slight deformation of CN rings at a high pressure of 5 GPa causes the splitting of the infrared peaks for the same vibration mode. Hence, at temperatures of up to 600 °C, the peaks in the wavenumber range of 1400–1700 cm^−1^ broaden significantly. The sample obtained at 700 °C shows a weak and featureless signal around 1630 cm^−1^ and 3440 cm^−1^ (see the zoomed spectrum in Figure S2), thereby indicating that the LTDP sample is likely to be an N-doped graphite [39].

**Figure 4:**
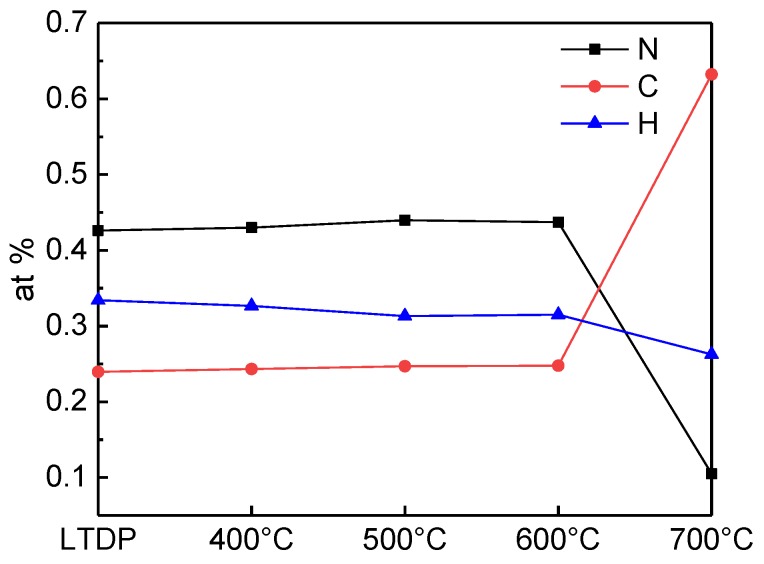
Elemental analysis of LTDP and the samples treated at 5 GPa and 400–700 °C.

Figure 4 shows the elemental analysis (EA) of the variation in carbon, nitrogen, and hydrogen contents with increasing pyrolysis temperatures. The nitrogen and carbon contents slightly increase, whereas the hydrogen content decreases as the pyrolysis temperature increases to 600 °C. This phenomenon is due to the polymerization of residual melamine in the sample. The dramatic increase in the carbon content occurs at 700 °C, indicating a high degree of graphitization. This finding is consistent with the observations in the XRD and FTIR spectra. The N/C/H atomic ratios of LTDP and the samples treated at 5 GPa and 400, 500, 600, and 700 °C are listed in Table S3.

## 4. Conclusions

This study investigates the high-pressure structural transition of melam at 5 GPa. Melam is synthesized through the multiple pyrolysis of melamine at 350 °C under nitrogen flow. During high-pressure processing, the structure of melam can be maintained on a preparative scale with a wide temperature range of 400–600 °C, and the content of residual or unreacted melamine decreases as temperature increases. No evidence of further melam polymerization is obtained before carbonization occurs at 700 °C. Our experimental findings reveal that the polymerization of C–N–H molecules is superior to their decomposition at high pressure. Thus, further polymerization of melam at a high pressure may be a promising strategy for the preparation of s-triazine-based carbon nitride precursors used for bulk CN synthesis.

## Figures and Tables

**Table 1:** Vibrational frequencies (cm^−1^) of melam, melamine, and LTDP

Vibrations	Melam (cm^−1^)	Melamine (cm^−1^)	LTDP (cm^−1^)
N–H	3483.8 (vw)	3468 (m)	3485 (vw)
	3456.5 (vw)	3420 (m)	3460 (w)
	3300.2 (w)	3331 (m)	3320 (w)
	3165.4(w)	3129 (m)	3182 (w)
Ring	1687 (vw)	-	-
	1639.5 (m)	-	-
	1610.0 (m)	-	-
	1583.8 (s)	1650 (vs)	1614 (vs)
	1545.8 (s)	1548 (vs)	1552 (m)
	1513.1 (s)	-	1515 (m)
	1450.8 (s)	1469 (vs)	1452 (vs)
	1414.7 (vs)	1438 (vs)	1429 (vs)
	1338.4 (vs)	-	1337 (vs)
	1249.8 (s)	-	1250 (m)
	1174.9 (w)	-	-
	1069.9 (vw)	-	-
	1021.1 (vw)	-	-
	972.2 (vw)	-	-
Out-of-Plane	806.2 (vs)	814 (vs)	808 (vs)
	782.1 (w)	-	-
	748.0 (vw)	-	-
	682.6 (m)	-	681 (w)

w: Weak; vw: Very weak; m: Middle; s: Strong; vs: Very strong.

## Supplementary Materials

The following are available online at http://www.mdpi.com/2079-4991/8/3/172/s1: Table S1: X-ray diffraction data of melamine; Table S2: X-ray diffraction data of melam cal.; Figure S1: Raman spectra of melam and the samples treated at 5 GPa and 400, 500, 600, and 700 °C at laser radiations of 325, 532, and 633 nm; Figure S2. FTIR spectrum of the sample treated at 5 GPa and 700 °C; Table S3: Elemental analysis of melam (cal. and exp.) and the samples treated at 5 GPa and different temperatures (400, 500, 600, and 700 °C).
