# Supplementary material for: Investigation on the Stability of Derivative Melam from Melamine Pyrolysis under High Pressure
Source: Nanomaterials (Basel). 2018 Mar 18;8(3):172. doi: 10.3390/nano8030172 (PMC5869663; doi:10.3390/nano8030172)
Supplement: Supplementary file 1 [file nanomaterials-08-00172-s001.pdf]

Supporting Information

Investigation on the stability of derivative melam from melamine pyrolysis under high pressure

Table S1. X-ray diffraction data of melamine.

h	k	l	d(Å)	2-theta	I/Imax
1	0	0	6.68	13.24	13.48
0	1	1	5.93	14.92	7.36
1	1	-1	5.05	17.56	5.69
1	0	-2	4.97	17.82	18.02
1	1	0	4.97	17.84	43.11
0	0	2	4.93	17.98	1.23
1	1	-2	4.13	21.49	1.54
0	1	2	4.11	21.62	34.53
1	1	1	4.00	22.18	33.54
0	2	1	3.47	25.62	2.38
2	0	-2	3.44	25.88	16.10
1	0	2	3.40	26.21	00.00
2	0	0	3.34	26.66	3.00
1	2	-1	3.27	27.28	20.58
1	2	0	3.25	27.46	2.16
1	1	2	3.09	28.88	44.80
0	1	3	3.01	29.70	36.73
1	2	1	2.93	30.53	2.40
1	0	-4	2.67	33.60	3.90
2	1	1	2.66	33.64	5.88
2	2	-1	2.59	34.64	1.53
1	2	-3	2.56	35.06	2.69
2	0	-4	2.49	36.10	5.81
2	2	0	2.48	36.14	7.40
0	2	3	2.46	36.48	7.43
1	1	3	2.44	36.78	1.20
3	0	-2	2.40	37.42	5.68
2	0	2	2.38	37.81	2.87
1	3	-1	2.33	38.63	10.49
3	1	-3	2.19	41.17	2.08
1	2	-4	2.17	41.68	1.56
1	2	3	2.12	42.58	1.22
3	1	-4	2.02	44.89	2.21
1	1	4	1.99	45.43	1.86
0	3	3	1.98	45.86	1.46
2	1	3	1.93	47.15	5.09
2	3	-3	1.90	47.75	7.52
2	3	1	1.87	48.68	3.38
3	1	-5	1.81	50.23	1.36
1	2	4	1.81	50.42	2.12
2	2	3	1.76	52.01	1.26
1	4	-2	1.74	52.59	1.11

4	0	-4	1.72	53.22	1.92
3	3	-1	1.71	53.42	1.51
4	1	-1	1.71	53.43	2.34
1	1	5	1.68	54.67	4.36
4	1	-4	1.68	54.75	1.59
3	0	-6	1.66	55.38	2.55
2	1	4	1.66	55.44	5.89
2	4	-1	1.65	55.64	1.55
1	4	-3	1.64	55.93	2.00
2	4	0	1.62	56.68	3.19
3	2	2	1.61	57.07	1.44
1	3	-5	1.61	57.11	2.12

Table S2. X-ray diffraction data of melam cal.

h	k	l	d(Å)	2-theta	I / I max
1	1	-1	7.94	11.13	6.15
1	1	1	7.54	11.73	2.28
2	0	-2	5.82	15.21	12.85
0	2	0	5.43	16.30	6.76
3	1	0	5.25	16.87	26.06
2	0	2	5.23	16.94	2.88
3	1	-1	5.08	17.45	5.31
0	2	1	5.06	17.51	12.90
2	2	0	4.65	19.06	3.81
4	0	0	4.50	19.71	2.65
2	2	-1	4.49	19.76	2.47
3	1	-2	4.40	20.17	2.16
2	2	1	4.34	20.46	3.04
1	1	3	4.06	21.89	30.25
3	1	2	4.01	22.16	1.76
4	0	-2	3.98	22.31	2.68
2	2	-2	3.97	22.37	2.30
2	2	2	3.77	23.59	0.76
3	1	-3	3.65	24.34	1.67
4	0	2	3.60	24.70	1.26
1	3	0	3.55	25.06	1.60
0	0	4	3.47	25.61	3.12
4	2	0	3.47	25.68	4.92
1	3	-1	3.46	25.74	5.61
5	1	-1	3.40	26.17	4.38
2	0	-4	3.37	26.44	46.57
5	1	1	3.24	27.50	3.90
4	2	-2	3.21	27.75	2.33
1	1	4	3.20	27.89	100.00
2	2	3	3.19	27.92	3.65
1	3	2	3.13	28.45	4.67
2	0	4	3.13	28.51	16.07
3	1	-4	3.04	29.40	4.65
5	1	2	2.95	30.30	1.66
0	2	4	2.93	30.51	3.98
5	1	-3	2.90	30.82	1.05
4	2	-3	2.90	30.86	3.45
1	1	-5	2.71	33.08	1.96
4	2	3	2.67	33.55	1.25
6	2	-1	2.63	34.10	1.19
6	2	0	2.63	34.11	1.25
5	1	-4	2.57	34.82	2.22
3	1	-5	2.56	35.00	5.36
5	3	0	2.55	35.11	1.04
6	2	1	2.54	35.36	1.37
0	4	2	2.53	35.45	4.49
6	2	-3	2.39	37.68	3.09
6	2	2	2.38	37.73	2.28
3	1	5	2.36	38.02	1.78

2	0	-6	2.31	39.04	1.05
1	1	-6	2.28	39.52	2.32

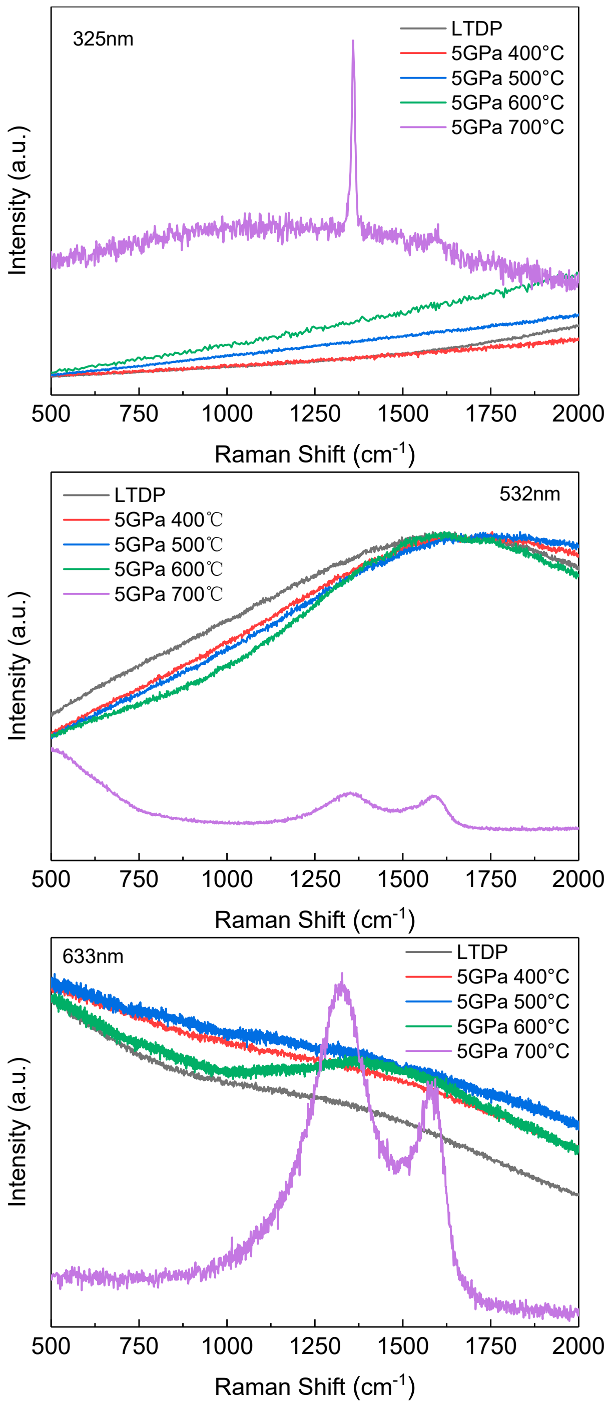

Figure S1. Raman spectra of LTDP and the samples treated at 5 GPa and 400 °C, 500 °C, 600 °C, and 700 °C at laser radiations of 325, 532, and 633 nm.

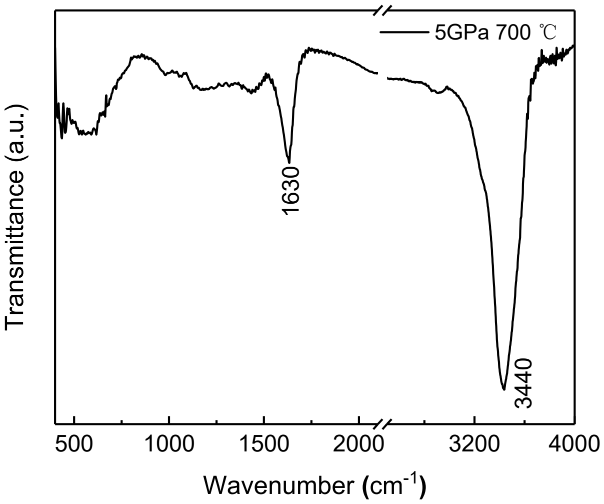

Figure S2. FTIR spectrum of the sample treated at 5 GPa and 700 °C.

Table S3. Elemental analysis of melam cal., LTDP and the samples treated at 5 GPa and different temperatures (400, 500, 600 and 700 °C).

Samples	N (at. %)	C (at. %)	H (at. %)
Melam cal.	42.31	23.08	34.62
LTDP	42.61	23.97	33.42
5GPa 400°C	43.01	24.33	32.67
5GPa 500°C	43.97	24.70	31.33
5GPa 600°C	43.72	24.77	31.51
5GPa 700°C	10.49	63.24	26.27
